# Generation of Functional Neuromuscular Junctions from Human Pluripotent Stem Cell Lines

**DOI:** 10.3389/fncel.2015.00473

**Published:** 2015-12-08

**Authors:** Katja A. Puttonen, Marika Ruponen, Nikolay Naumenko, Outi H. Hovatta, Pasi Tavi, Jari Koistinaho

**Affiliations:** ^1^Department of Neurobiology, A.I. Virtanen Institute for Molecular Sciences, University of Eastern FinlandKuopio, Finland; ^2^School of Pharmacy, University of Eastern FinlandKuopio, Finland; ^3^Department of Biotechnology and Molecular Medicine, A.I. Virtanen Institute for Molecular Sciences, University of Eastern FinlandKuopio, Finland; ^4^Department of Clinical Science, Intervention and Technology, Karolinska InstitutetStockholm, Sweden

**Keywords:** induced pluripotent stem cell, embryonic, human, cell culture, nerve cell engineering, skeletal muscle

## Abstract

Several neuromuscular diseases involve dysfunction of neuromuscular junctions (NMJs), yet there are no patient-specific human models for electrophysiological characterization of NMJ. We seeded cells of neurally-induced embryoid body-like spheres derived from induced pluripotent stem cell (iPSC) or embryonic stem cell (ESC) lines as monolayers without basic fibroblast factor (bFGF) and observed differentiation of neuronal as well as spontaneously contracting, multinucleated skeletal myotubes. The myotubes showed striation, immunoreactivity for myosin heavy chain, actin bundles typical for myo-oriented cells, and generated spontaneous and evoked action potentials (APs). The myogenic differentiation was associated with expression of MyoD1, myogenin and type I ryanodine receptor. Neurons formed end plate like structures with strong binding of α-bungarotoxin, a marker of nicotinic acetylcholine receptors highly expressed in the postsynaptic membrane of NMJs, and expressed SMI-32, a motoneuron marker, as well as SV2, a marker for synapses. Pharmacological stimulation of cholinergic receptors resulted in strong depolarization of myotube membrane and raised Ca^2+^ concentration in sarcoplasm, while electrical stimulation evoked Ca^2+^ transients in myotubes. Stimulation of motoneurons with N-Methyl-D-aspartate resulted in reproducible APs in myotubes and end plates displayed typical mEPPs and tonic activity depolarizing myotubes of about 10 mV. We conclude that simultaneous differentiation of neurons and myotubes from patient-specific iPSCs or ESCs results also in the development of functional NMJs. Our human model of NMJ may serve as an important tool to investigate normal development, mechanisms of diseases and novel drug targets involving NMJ dysfunction and degeneration.

## Introduction

Disorders of the neuromuscular junctions (NMJs), the cholinergic synapses between lower motoneurons and skeletal muscle fibers, include myasthenia gravis (MG) and Lambert-Eaton myasthenic syndrome (LEMS; Liang and Han, 2013). In addition, recent studies indicate that progressive paralyzing diseases, amyotrophic lateral sclerosis (ALS; Maselli et al., 1993; Frey et al., 2000; Das et al., 2010; Wu et al., 2010; Naumenko et al., 2011; Palma et al., 2011; Thomson et al., 2012) and spinal muscular atrophy (SMA; Russman, 2007), involve early pathological changes at NMJ. While all these disorders belong to the class of orphan (rare) diseases, their prevalence altogether is 100,000—130,000 in the US, 200,000—260,000 in Europe, and approximately 1.5–1.8 million worldwide (http://www.rightdiagnosis.com/n/neuromuscular_conditions/prevalence.htm). These diseases are a serious global concern as there is no known cure for SMA (Monani and De Vivo, 2014) or ALS (Gordon, 2013) and the currently available therapies for MG and LEMS can cause severe adverse effects without providing sufficient benefit for all patients.

Even though the structure and function of NMJ are well characterized, the current understanding of the NMJ is largely based on findings using animal models and animal cells (reviewed in Wu et al., 2010; Thomson et al., 2012). The lack of human models of human diseases, especially of neurodegenerative and muscle diseases, is considered to be a major reason for the fact that hundreds of therapies that show excellent promise in preclinical studies do not translate to clinics (Palma et al., 2011; Sleigh et al., 2011; Boltze et al., 2012; Thomson et al., 2012). Therefore, there is an urgent need for development of completely human-based NMJ models for unraveling the molecular mechanism and therapeutic possibilities for treating diseases involving synapse pathology, and especially the diseases with NMJ dysfunctions. Significant progress has been made by studies where motoneurons and muscle cells for a co-culture were derived from human fetal spinal stem cells and adult muscle stem cells, respectively (Guo et al., 2011). However, for modeling diseases of genetic etiology an NMJ model where both neuron and muscle components are derived from the same individual is of great interest.

Availability of human pluripotent stem cells (hPSC), either of embryonic (ESC), or induced pluripotent (iPSC) origin, has made the generation of human muscle and nerve cells possible. Several reports of hPSC differentiation into myotubes (i.e., precursors of muscle fibers; Barberi et al., 2005, 2007; Zheng et al., 2006; Mahmood et al., 2010; Darabi et al., 2011; Awaya et al., 2012; Goudenege et al., 2012; Ryan et al., 2012; Abujarour et al., 2014; Hosoyama et al., 2014) and motoneurons (Hu and Zhang, 2009; Nizzardo et al., 2010; Hester et al., 2011; Qu et al., 2014) have been published. Also, hPSC-derived motoneurons have been co-cultured with rodent muscle cells or muscle cell lines but not with muscle cells originating from cells of human embryos (ESCs) or adult patients (iPSCs; Das et al., 2010; Guo et al., 2011). Such an ESC or iPSC-derived model of NMJ would be useful, allowing the neuronal and muscle components of the NMJ to be derived from the same individual (reviewed in Thomson et al., 2012). Moreover, especially human iPSC-derived NMJ models would provide new insights into the mechanisms of genetic diseases involving NMJ dysfunction, help patient stratification, and further enable the design of personalized therapy.

Production of highly pure population of the final cell types, e.g., specific types of neurons or myofibers, has been one of the major obstacles in the differentiation processes of hPSCs (Chiu and Rao, 2011; Salani et al., 2012). While high purity of the final cell type is indeed desired for many experimental set-ups, such as cell-specific expression analyses of genes or proteins, most of the cellular functions and disease pathologies depend on several cell types and their interaction. This view is valid also for the diseases involving NMJ dysfunction, such as ALS, SMA, MG, and LEMS (Palma et al., 2011; Thomson et al., 2012; Liang and Han, 2013). Therefore, the cell culture models of NMJ in which neurons and muscle cells develop in interaction with each other might be the most relevant ones. Although hiPSC-derived cell models are not likely to reach fully mature phenotype in culture *in vitro* (Yoshida et al., 2015), the models with functional NMJs would be valuable in studying development of pathology associated with genetic disorders and developmental assembly of human NMJs in general.

We have earlier reported that the human ESCs differentiated into neural progenitors (NPCs) in a suspension produce small spindle-shaped cells that further differentiate into neural cells, and large flat cells that are negative for a variety of neuronal and glial markers (Puttonen et al., 2013). Here we report that a portion of the non-neural flat cells differentiate into myogenic cells and form multinucleated skeletal myotubes in the environment designed for neural induction, proliferation and maturation. Importantly, the presence of progenitors for both neurons and skeletal muscle cells allow co-maturation of these two cell types, resulting in formation of functional NMJs. Therefore, by taking advantage of the apparent developmental interdependency of skeletal muscle cells and motoneurons, and their parallel derivation from hPSCs of the very same individual, our novel model may serve as an important tool to investigate normal development and mechanisms of diseases involving NMJ dysfunction and degeneration.

## Materials and methods

### Maintenance of human PSC cultures

Human ESCs (line HS306, originally derived at Karolinska Institutet, Stockholm, Sweden) and hiPSCs (lines UEFhfiPS1.4 and UEFhAD2fiPS1.2) reprogrammed and characterized in our laboratory (Qu et al., 2013, unpublished data) were cultured as previously described (Puttonen et al., 2013). Ethics approval (No: 42/2010) was obtained by Pohjois-Savon Sairaanhoitopiirin kuntayhtymä, Tutkimuseettinen toimikunta, and individuals donating skin samples gave informed consent before taking part in the study.

### Differentiation of embryoid body-like spheres and induction of maturation

Neural differentiation of HS306, UEFhfiPS1.4 and UEFhAD2fiPS1.2 lines was performed as previously described (Puttonen et al., 2013) except that at the end of the differentiation period (>6 weeks after induction of differentiation) and prior to plating down the individual cells, the spheres were not selected for the experiments but all types and sized spheres were collected. Thus, we ensured that not only neurospheres and neural progenitors but also heterogeneous embryoid body (EB) –like large spheres containing non-neuronal cells were included (Puttonen et al., 2013). Terminal maturation of the cells was performed as described (Puttonen et al., 2013). Briefly, dissociated progenitor cells (1 × 10^5^ cells/cm2) were seeded on polyornithine and laminin-coated wells in the medium containing 1:1 mix of DMEM/F12 and Neurobasal media, 1x retinoic acid free B27, 1x N2, 2 mM Glutamax, 50 IU/ml Penicillin and 50 μg/ml Streptomycin (all from Invitrogen, Carlsbad, CA), and maintained at 37°C and 5% CO_2_ for 5–7 days until performing analyses. Medium was changed every second day. For some batches the terminal maturation phase was recorded by a continuous live cell imaging system, Cell-IQ v.2, equipped with a phase-contrast microscope and a 10X objective (Chip-Man Technologies, Tampere, Finland). The plates of monolayered cells were covered with a Cell-Secure lid (Chip-Man Technologies) containing ventilation filters. The incubation conditions were maintained at 37°C, 5% CO_2_, 19% O_2_, and 76% N2. The medium was changed every other day. The culture was time-lapse imaged every 30 min for 6 days with an Imagen™ Mode. Image selection and video reconstruction was made by an Analysis™ Mode.

### Labeling of cells for fluorescence microscopy

Immunocytochemical labeling was performed as described (Puttonen et al., 2013) for all cell lines/differentiation batches. Primary antibodies were anti-doublecortin (DCX; 1:400; 4604, Cell Signaling Technology, Danvers, MA) and anti-βIII-tubulin (Tuj1; 1:1000; MMS-435P; Covance, San Diego, CA) for neurons, Neurofilament H Non-Phosphorylated (SMI 32) Monoclonal Antibody (1:1000; SMI-32P; Covance, Princetown, NJ) for motoneurons [36], anti-synaptic vesicles (SV2; 1:100; Developmental Studies Hybridoma Bank, Iowa, IA) and anti-Synaptophysin (anti-Synaptophysin; 1:100; Abcam, 14692, Cambridge, UK) for synapses, and anti-Myosin heavy chain (MHC clone A4.1025; 1:400; 05-716; Millipore, Billerica, MA) for myotubes. Alexa Fluor® goat anti-mouse and anti-rabbit 488 and 568 (1:300; Molecular Probes®, Invitrogen) were used as the secondary antibodies. Acetylcholine receptors of NMJs were visualized by staining with 0.25–1 μM Alexa Fluor 488 or 594 α-bungarotoxin (B13422 and B13423, Molecular Probes®, Invitrogen). Alexa Fluor 488 Phalloidin (25 nM; Molecular Probes®, Invitrogen) was used for labeling F-actin in microfilaments to visualize the finest cytoskeletal organization of the myogenic cells. Fluorescently labeled cultures were imaged and analyzed with an Olympus DP70 digital camera attached to an Olympus IX71 inverted epifluorescence microscope (Hamburg, Germany) or with a FluoView 1000 (Olympus, Japan) confocal microscope.

### Quantitative RT-PCR (qRT-PCR)

The extraction of total RNA, reverse transcription, and qRT-PCR analyses were performed as described earlier (Puttonen et al., 2013). TaqMan gene expression assays (Applied Biosystems) containing FAM dye were used to measure the RNA expression of microtubule-associated protein (MAP2, Hs00258900_m1), paired box protein (PAX6, Hs00242217_m1), myogenic differentiation 1 (MyoD1, Hs02330075_g1), myogenin (MYOG, Hs01072232_m1) and ryanodine receptor 1 (RYR1, Hs00166991_m1) genes. For each sample the expression of endogenous β–actin ACTB gene containing VIC-dye (Applied Biosystems) was determined and used for normalization.

### Ca^2+^ imaging and patch-clamp recordings

For Ca^2+^ imaging the attached cells were loaded with 5 μM Fluo-4-acetoxymethyl (AM)-ester (Invitrogen) at 37°C for 30 min, and then placed in the recording chamber continuously perfused with preheated (37°C) Dulbecco's modified Eagle medium containing glutamax I (DMEM; Invitrogen) and bubbled with 95% O_2_ and 5% CO_2_. To excite the cells, a stimulator (Grass, USA) and platinum wires were used. In some experiments acetylcholine (100 μM, Sigma) was added in bath solution to activate nicotinic receptors. Ca^2+^ imaging was performed with a confocal inverted microscope (FluoView 1000, Olympus). The cells were excited at 488 nm and the emitted light band was set at 500–600 nm. The fluorescence intensity is expressed as an F/F_0_ -ratio, where F is the background subtracted fluorescence intensity and F_0_ is the background subtracted minimum fluorescence value measured from each cell at rest. The electrical activity and action potentials (APs) were measured in the same chamber by conventional whole-cell patch recording in a current clamp mode (Axopatch 200B, Digidata 1440A, Molecular Devices Inc., USA). Patch electrodes (3–4 MΩ) made of borosilicate glass (Harvard Apparatus, UK) were filled with (mM): 120 K-aspartate, 25 KCl, 1 MgCl2, 2 Na2-phosphocreatine, 4 Na2-ATP, 2 NaGTP, 10 EGTA, and 5 HEPES, pH 7.2 with KOH [36]. Evoked APs were elicited by a 1 ms current injection. For chemical stimulation carbacholine (100 μM, Sigma) or NMDA (500 μM, Sigma) were applied directly to the studied area with local perfusion manifold (Cell MicroControls, USA).

### Statistics

The statistical significance of the data was analyzed with paired *T*-test using GraphPad Prism version 5 (GraphPad Software, Inc., San Diego, CA). *P* < 0.05 were considered statistically significant.

## Results

### Differentiation of myotubes in neural cell co-cultures

**Figure 1:**
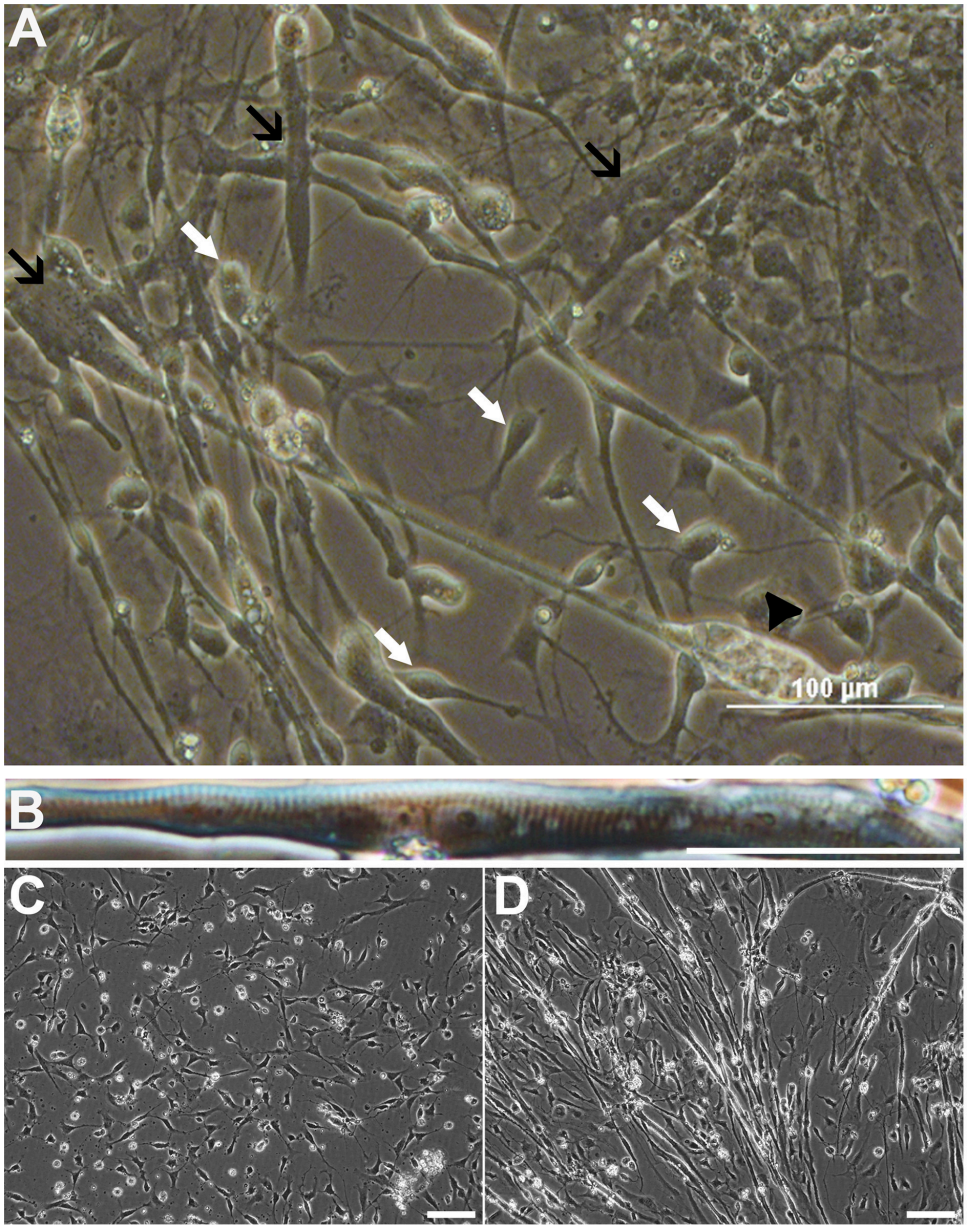
**Cells from non-selected spheres mature into neurons and myotubes derived from ESCs (HS306)**. Cell populations contain neural progenitors and mature neurons with neurites (**A**, white arrows), and non-striated (**A**, black arrows) and striated **(B)** multinucleated myotubes. In these cultures, myotubes are spontaneously contracting **(A**, Video S1**)**. Eventually, the contraction and movement may lead to the detachment and further degeneration of the myotube (**A**, black arrowhead). Time-lapse imaging of the same view reveals that myoprecursors and myotubes are highly mobile, and the fusion of precursor cells into myotubes is a dynamic process **(C,D**, Video S2**)**. Scale bars: **(A)** 100 μm; **(B)** 50 μm; **(C,D)** 100 μm.

To investigate the differentiation fate of the non-neural cells, we seeded cells from all sphere types derived from iPSC or ESC lines onto polyornithine-laminin coated wells as monolayers, and withdrew basic fibroblast factor (bFGF) from the growth medium. As expected, 7 days later cells with a typical NPC morphology were seen (Figure 1A, white arrows). However, we also observed a large number of spontaneously contracting (Video S1), multinucleated cells (Figures 1A,B). Some of these cells exhibited striation characteristics for skeletal muscle cells (Figure 1B). During the terminal differentiation phase, continuous live cell imaging revealed spontaneous fusion of muscle precursors and subsequent formation of myotubes (Figures 1C,D; Video S2). Notably, the mature myotubes tended to detach from the culturing plate due to their twitching.

**Figure 2:**
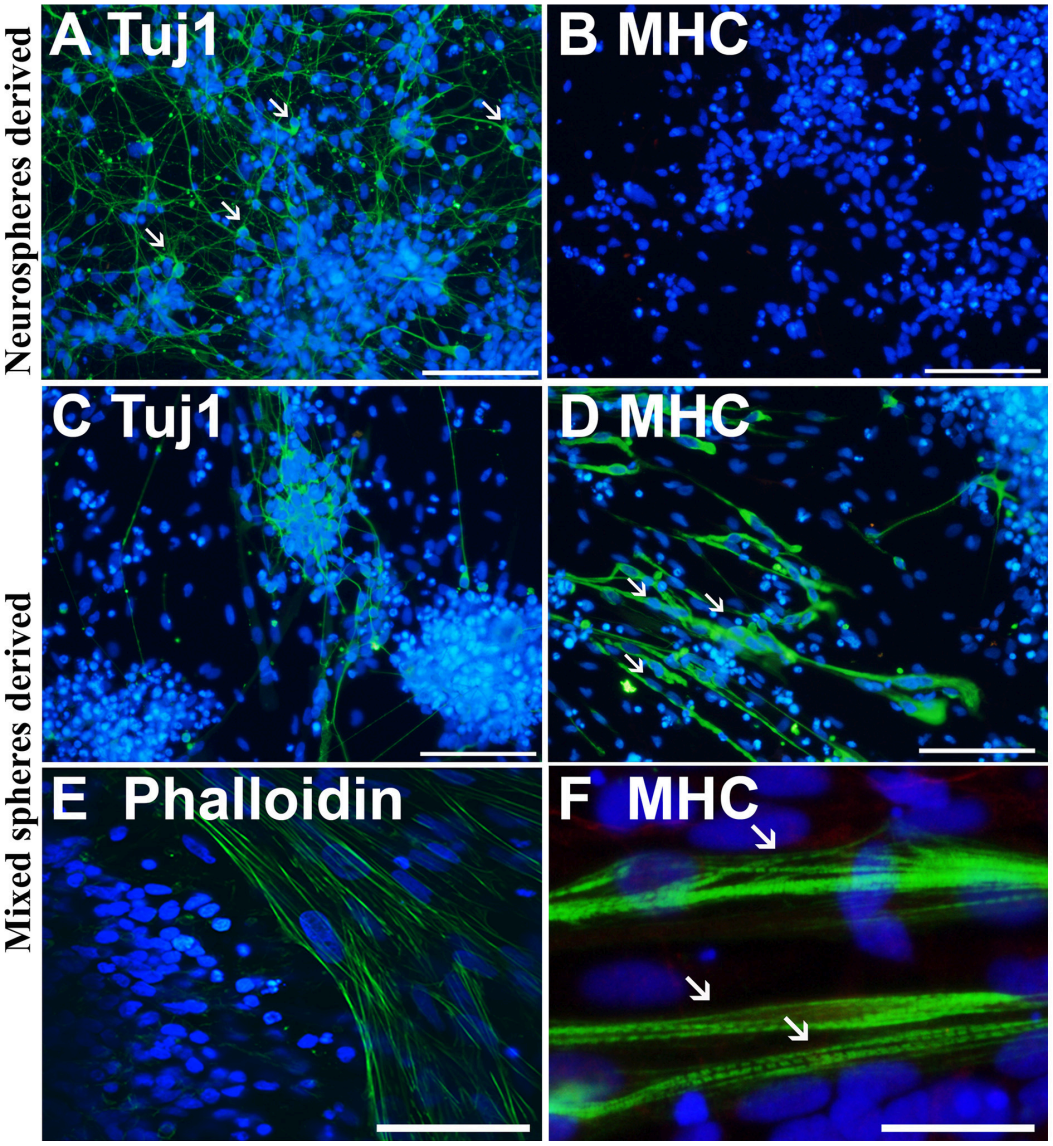
**Immunostaining confirms that the cells from non-selected spheres mature into neurons and myotubes**. Neurosphere-derived cells mature predominantly (>90%) into neuron specific, class III β-tubulin (Tuj1) positive neurons with small nuclei and long projections (**A**, white arrows). These cells do not express a skeletal muscle marker myosin heavy chain (MHC) **(B)**. Non-selected (mixed) spheres produce both neurons **(C)** and thick MHC positive **(D)** myotubes (**D**, white arrows) that are also positive to phalloidin **(E)**, labeling the actin filaments of myo-oriented cells. In some MHC positive myotubes, the striation is visible (**F**, white arrows). In **(A–F)** the nuclei were stained with Hoechst 333042 (blue). Scale bars: **(A–D)** (iPSC-derived), 100 μm; **(E)** (ESC-derived), 50 μm; **(F)** (iPSC-derived), 30 μm.

**Figure 3:**
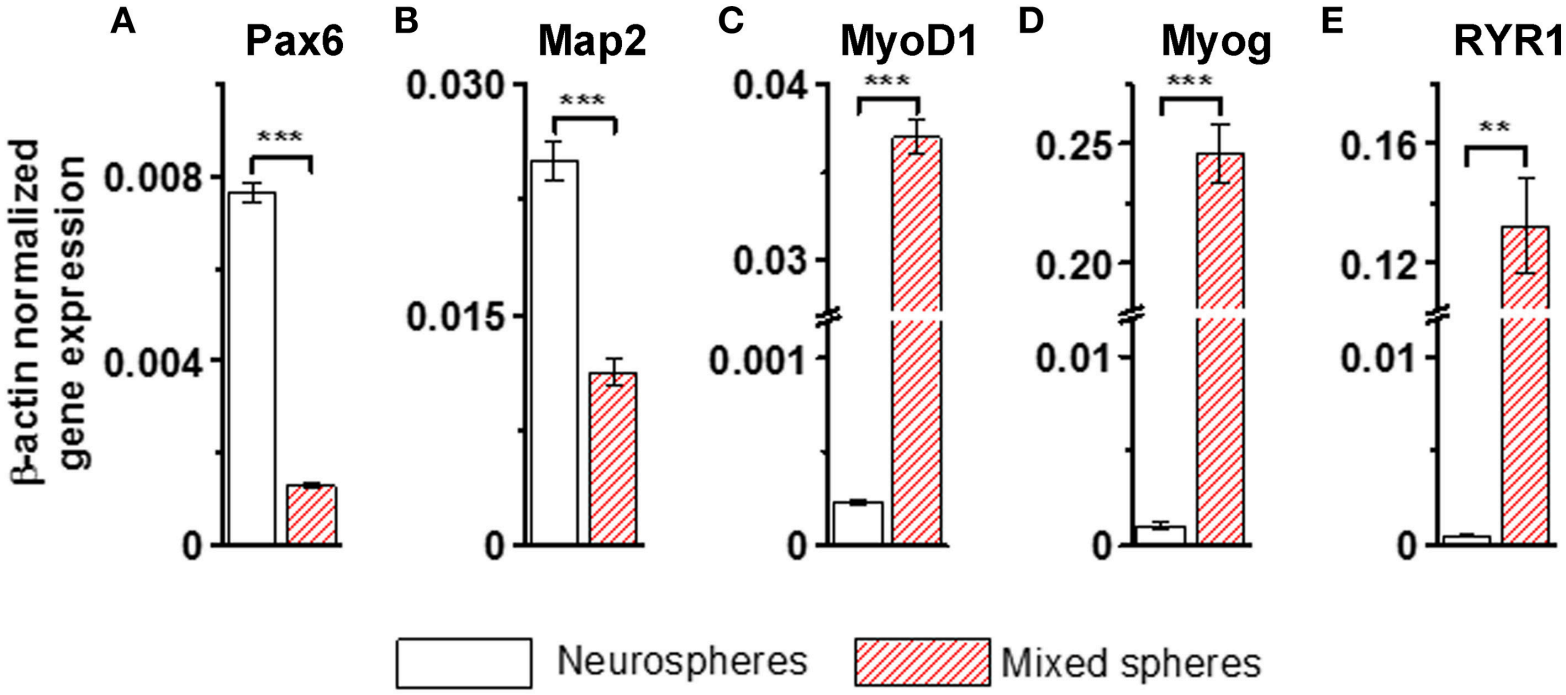
**Neural genes (A,B) are strongly down-regulated and myogenic markers (C–E) up-regulated in the non-selected (mixed) sphere-derived cell populations when compared to neurospheres**. Results are shown as the normalized expression of the target genes against the expression of the endogenous β-actin gene. Results are expressed as mean ± *SD*, *n* = 4, and analyzed with paired *T*-test, ^**^ <0.005, ^***^ <0.001. Map-2, microtubule- associated protein; MyoD1, myogenic differentiation 1; Myog, myogenin; Pax-6, paired box protein; RYR1, ryanodine receptor 1. All cells were derived from iPSCs.

Immunofluorescence labeling with anti-neuron specific class III β-tubulin (Tuj1) and anti-MHC, a marker of muscle cells, confirmed that purely neurally oriented cultures seeded from selected spheres lacked myogenic, MHC-immunoreactive cells (Figures 2A,B). Instead, in cultures derived from all types of spheres maturation of both neuronal (Tuj1-immunoreactive) and myogenic (MHC-immunoreactive) markers was seen (Figures 2C,D). In addition, Phalloidin 488 staining of the myotube-like cells revealed actin bundles typical for myo-oriented cells (Figure 2E) and in some MHC-immunostained myotubes the striation was visible (Figure 2F). Finally, quantitative RT-PCR demonstrated that the expression of neuroectodermal (Pax-6) and neuronal (Map-2) genes was several fold lower in myotube-containing cultures compared to cells of neuron-enriched batches (Figures 3A,B). Correspondingly, the expression of MyoD1, a master gene of myogenic process, and the two genes describing the terminal maturation of myotubes, Myogenin and type I Ryanodine receptor (Goudenege et al., 2012; Tran et al., 2013) were significantly, more than 160-fold, higher in cultures containing myotubes compared to the differentiation batches that were purely neural (Figures 3C–E).

### Cytochemical identification of NMJ

Having shown the co-maturation of neurons and myotubes in our cell culture model of hPSC-derived cells, we next investigated whether the two cell types are synaptically connected to each other. NMJ is a special type of synapse in which the AP of a motoneuron leads to activation (contraction) of innervated muscle fibers (Das et al., 2010; Wu et al., 2010).

**Figure 4:**
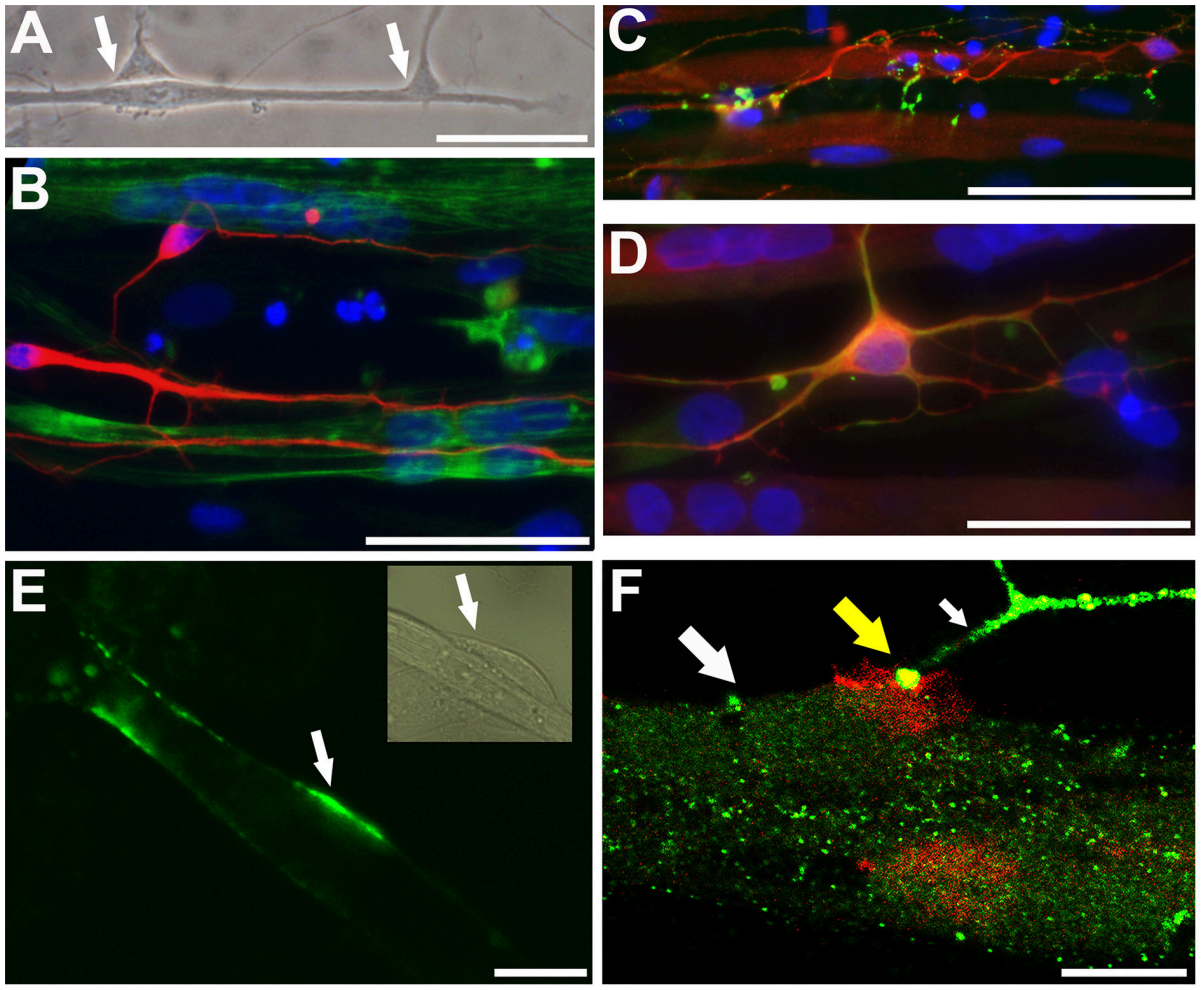
**Neurons and myotubes that are in close proximity seem to connect with each other potentially via neuromuscular junction (NMJ)**. A representative phase contrast image (**A**; ESC-derived) shows an elongated muscle cell contacting with two neuronal cells (arrows). Immunocytochemistry shows that neuron specific III β-tubulin (Tuj1) positive neuronal cells (**B**; iPSC-derived, in red) grow along and surround the phalloidin positive myotubes (in green). A proportion of doublecortin (DCX) positive neurons (**C**; iPSC-derived, in red; also myotubes express DCX) express a synaptic protein, synaptic vesicles (SV2; **C** in green). In **(D)** (iPSC-derived), DCX positive neurons (in red) contacting with multinucleated myotubes co-express (in yellow) neurofilament H (SMI-32; in green), a motoneuronal protein. In **(E)** (ESC-derived), α-bungarotoxin (in green) specifically binding to the nicotinic acetylcholine receptors densely expressed at the postsynaptic site of NMJ is detected on the surface of myotubes (the phase contrast insert shows presumably a neuron contacting the myotube). In **(F)** (confocal image, iPSC-derived), a thick myotube (big white arrow) expressing post-synaptic membranes, visualized with red-labeled α-bungarotoxin, is contacting with a synaptophysin positive nerve ending (green; small white arrow). At the site of NMJ the colocalization is seen (in yellow color; yellow big arrow). In **(B–D)** the nuclei were stained with Hoechst 33042 (blue). Scale bars: **(A–D)**, 50 μm; **(E,F)**, 10 μm.

Phase-contrast microscopy and immunocytochemical analyses showed intimate connections between neuronal processes and myotubes derived from iPSC and ESC lines (Figure 4A). Neuronal processes immunoreactive for neuron-specific type III β-tubulin (Tuj1) were observed to surround phalloidin positive myotubes derived from either iPSC or ESC lines (Figure 4B). Varicose doublecortin immunoreactive nerve fibers on myotubes expressed also synaptic vesicle protein 2 (SV2), a membrane glycoprotein found in the secretory vesicles of neural cells (Custer et al., 2006; Figure 4C). Moreover, in line with the idea of NMJ formation in these cultures, a portion of the neurons expressed a non-phosphorylated epitope in neurofilament H (SMI-32), a motoneuron marker (Figure 4D). Finally, when NMJs were labeled with fluorogenic α-bungarotoxin, a specific marker of nicotinic acetylcholine receptors (nAChR) known to be densely expressed in the NMJ postsynaptic membrane (Bruneau et al., 2005), bright fluorescence was observed at end plates (Figure 4E) and colocalized with synaptophysin immunoreactivity (Figure 4F). These results led us to hypothesize that both the neuronal cells and the postsynaptic membranes present in myotubes of our human co-culture model are mature enough to form functional NMJs.

### Functional properties of myotubes and NMJs

**Figure 5:**
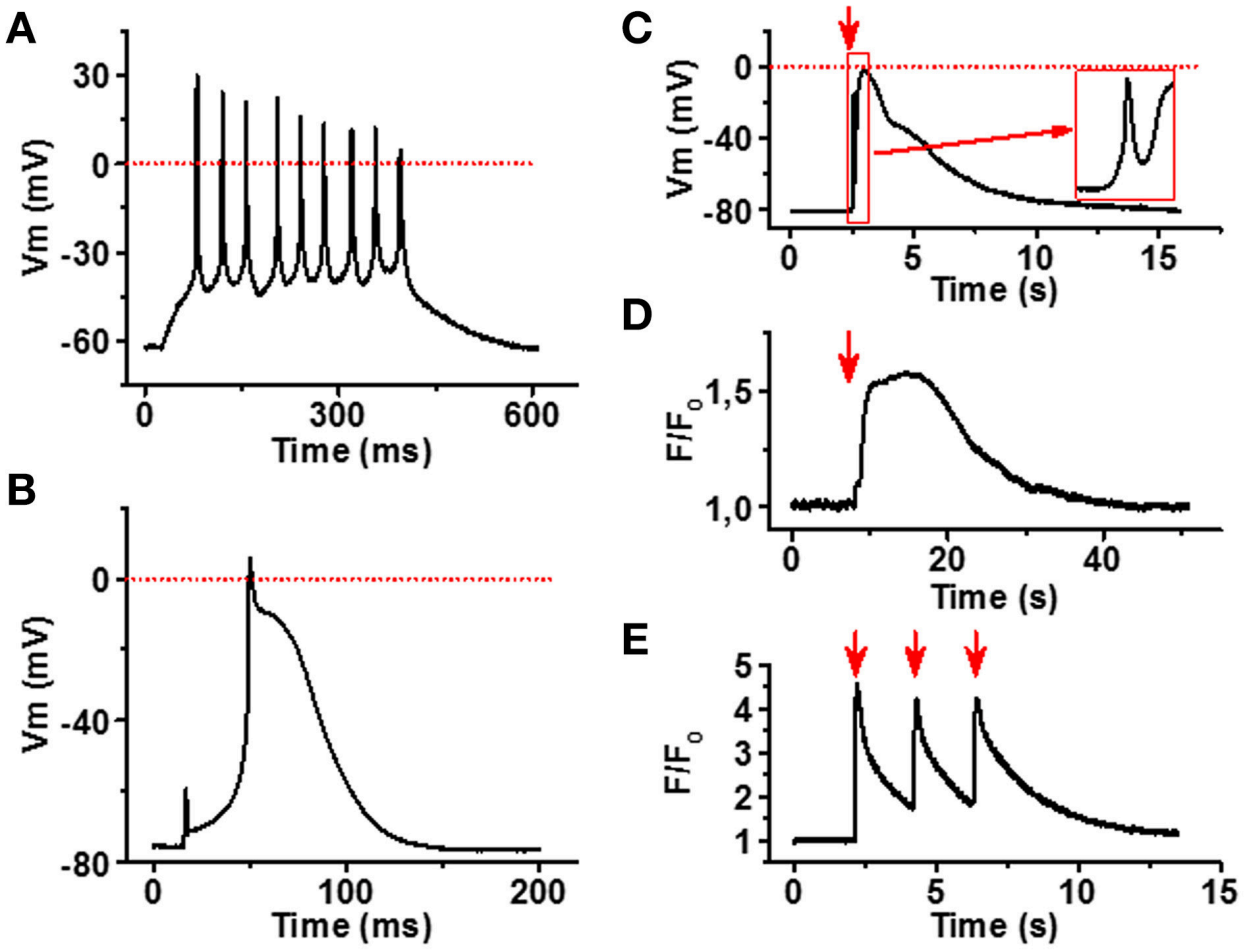
**Functional characterization of iPSC-derived myotubes**. **(A)** Membrane potential (Vm) recording of spontaneously active myotube showing repetitive firing and narrow spike-like APs. **(B)** In part of the myotubes current injection evokes a single relatively long AP. **(C)** Carbacholine application (red arrow) causes strong depolarization of the myotube membrane. Note the fast phase (inset) most likely induced by the current through nAChR. **(D)** Acetylcholine (100 μM) application (red arrow) induces a robust increase in cytosolic [Ca^2+^] of the myotubes, whereas **(E)** 0.5 Hz electrical stimulation (red arrows) evokes repetitive calcium transients in myotubes. All traces are representative examples out from at least 15 recordings.

To confirm that the myotubes and NMJs identified in our co-culture model are functional, we applied electrophysiology and live cell Ca^2+^ imaging to characterize the spontaneous and induced myotube responses. As expected, myotubes had an ability to generate spontaneous and evoked APs (Figures 5A,B). Myotubes had relatively low resting membrane potential (65–80 mV) and the AP waveform was variable, especially the AP duration showed large variability ranging from relatively narrow spike-like AP to ones that lasted up to 200 ms. The finding indicates that among cells showing myotube morphology the degree of maturation differed from cell to cell.

To examine functionality of our NMJ model, we first stimulated the cultures with carbacholine, a cholinomimetic that binds and activates nAChRs in the NMJ postsynaptic membrane (Nachev et al., 2008). At 100 μM concentration, carbacholine produced fast and strong depolarization of the myotube membrane (Figure 5C), in line with the localization of dense α-bungarotoxin binding observed in end plate-like structures. To see if membrane depolarizations trigger muscle-type cytosolic calcium signals (Hernández-Ochoa and Schneider, 2012), we measured intracellular Ca^2+^ signals in fluo-4 loaded cells after electrical field stimulation of myotubes. As seen in Figure 5E, 0.5 Hz electrical stimulation of myotubes evoked strong Ca^2+^ transients in myotubes. Moreover, application of 100 μM acetylcholine induced robust calcium elevation in sarcoplasm (Figure 5D).

**Figure 6:**
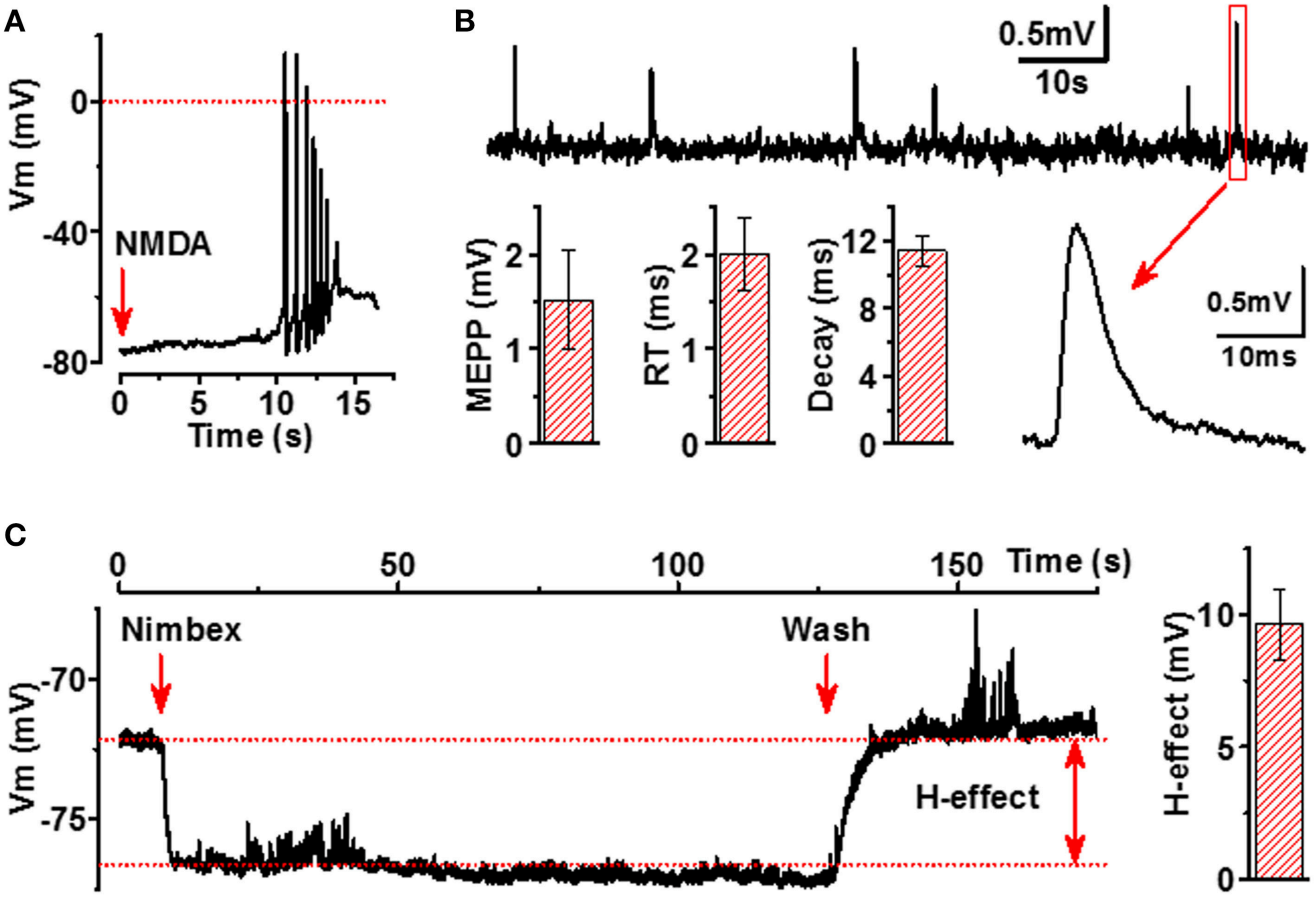
**Functional characterization of NMJs in iPSC-derived cells**. **(A)** Activation of NMDARs in neurons by application of NMDA (500 μM, red arrow) induces AP firing of adjacent myotubes. **(B)** Membrane potential recording of miniature end-plate potentials (mEPPs) of myotubes (upper line), example of expanded mEPP trace (lower line, right part) and main characteristics of mEPP (lower line, left part, mEPP: amplitude, rise time (RT) and decay of mEPP). **(C)** Hyperpolarizing effect (H-effect) of nimbex (2 μM) a nicotinic receptor blocker on resting membrane potential (application—the first red arrow, wash—the second red arrow). All traces are representative examples out from at least 8 recordings.

Next, we analyzed whether excitation of motoneurons results also in activation of myotubes, thereby indicating functionality of the NMJs. The most well-known excitatory receptors expressed by motoneurons but not by myotubes are glutamatergic receptors, especially N-Methyl-D-aspartate (NMDA) receptors (Vartiainen et al., 1999). Thus, we applied micromolar concentrations of NMDA to our co-culture model and observed reproducible APs in myotubes, confirming that neurons contacting myotubes are both presynaptically and postsynaptically mature and that NMJs between NMDA-activated neurons and myotubes are truly functional (Figure 6A).

Spontaneous ACh release into synaptic cleft accomplished by both quantal and non-quantal manner (Katz and Miledi, 1977) is an essential feature of NMJs. A single quantum of ACh brings about the appearance of one miniature end-plate potential (mEPPs), whereas non-quantal secretion of ACh results in a tonic effect on postsynaptic resting membrane potential (Stanley and Drachman, 1979; Urazaev et al., 2000). As seen in Figure 6B, the human myotubes in our culture model generated mEPPs with characteristics completely matching to those of the mEPPs previously reported for primary skeletal muscle cells (Santafé et al., 2003). When analyzing 330 myotubes from 30 different culture wells, 34 functional NMJs with detectable and reliable mEPP were identified. Considering that mEPPs cannot be expressed without functional presynaptic and postsynaptic counterparts of NMJs, our data indicate that at least 300 functional NMJs per 300 000 cells in each culture wells were present. Moreover, typical to innervated muscle cells, the myotubes in our cell culture model demonstrated fast and strong hyperpolarization (9.6 ± 1.3 mV, *n* = 8) upon a local application of nicotinic receptor blocker (Figure 6C), a phenomenon known to occur in innervated but not in non-innervated muscle cells (Sun and Poo, 1985). Altogether, our electrophysiological results indicate that ACh is spontaneously released into NMJs by both quantal and non-quantal manner in our cell culture model.

## Discussion

We have reported earlier that a large amount of NPCs can be produced from hPSCs by growing cells in spheres in the neural induction and proliferation medium (Puttonen et al., 2013). Yet, when no pre-selection methods are applied, the cultures produce not only neurospheres but also EB-like spheres that contain predominantly non-neural cells (Puttonen et al., 2013). Here we show that the EB-like spheres that are produced in addition to neurospheres from hESCs or hiPSCs (in floating aggregates) in neural proliferation medium give rise to myotubes and neurons, when the spheres are dissociated and maintained as adherent cultures after withdrawal of FGF. The data indicates that cells originating from a single individual can be differentiated into both neurons and myotubes in the same cultures when cells from all sized and heterogenous spheres are included in the terminal differentiation phase. This is in line with the previous reports demonstrating that the medium optimized for neural differentiation does not prevent the random formation of various types of spheres containing numbers of non-neural cells (Nat et al., 2007; Puttonen et al., 2013; Hosoyama et al., 2014). More importantly, our data shows that the culture medium containing FGF during the 6-week neural proliferation phase followed by brief neural differentiation phase as adherent cultures without FGF favors differentiation of both neurons and myotubes. While Hosoyama et al. (2014) reported that hPSC sphere cultures give rise to myogenic progenitor cells when hPSC colonies were expanded in the medium containing high, 100 ng/ml concentrations of FGF and EGF, our results show that the neural proliferation medium containing low concentration (25 ng/ml) of FGF is sufficient for myogenic differentiation without compromising the proliferation and differentiation of neuronal cells.

The molecular machinery required for synaptic transmission was present in post-synaptic membranes of myotubes developed in our co-culture system, as evidenced by the dense α-bungarotoxin binding at end plate-like structures, the fast and strong depolarization of myotubes induced by carbacholine, and transient Ca^2+^ signals induced by acetylcholine or in response to electrical stimulation. Even though the properties of the resting and action potential indicated that maturation phase of the myotubes varied from cell to cell, the postsynaptic membranes present in myotubes of our human co-culture model were mature enough to form functional NMJs. While there are no previous reports on electrophysiological properties of hPSC-derived myotubes available, our findings are in line with results obtained from cultured myotubes derived from human and mouse satellite cells (Das et al., 2010; Guo et al., 2011; Yoshida et al., 2015) and confirm that our hPSC-derived myotubes closely resemble myotubes that are obtained without genetic reprogramming and differentiated in co-culture with neurally oriented cells.

The finding of the non-quantal release of ACh into the NMJ clefts of our co-culture system can be considered to indicate active NMJ formation and maintenance (Fu et al., 1998). This notion is supported by the previous studies reporting that spontaneous ACh release appears at embryonic nerve cones prior to synapse formation (Young and Poo, 1983) and during the development of embryonic myotubes before they become innervated (Fu et al., 1998). Importantly, presynaptic nicotinic receptors and muscarinic receptors at nerve ending of developing motoneurons, which were identified also in our NMJ model, modulate the spontaneous release of ACh (Santafé et al., 2003). The mechanisms of how non-quantally released ACh may promote NMJ maturation and maintenance include its function as a specific chemoattractant for growth cone guidance (Zheng et al., 1994) and its contribution to the anterograde and retrograde signaling between presynaptic and postsynapic membranes (Dan and Poo, 1994; Urazaev et al., 2000), the latter being considered to be an interaction mandatory for the successful development and function of synapses (Katz and Miledi, 1977; Mathers and Thesleff, 1978; Bray et al., 1982; Young and Poo, 1983; Vyskocil and Vrbová, 1993; Dan and Poo, 1994; Fu et al., 1998; Urazaev et al., 2000). Because, non-quantal release is more pronounced in developing endplates than in fully mature end plates (Vyskocil and Vrbová, 1993), reflecting its important role in NMJ development overall, our cell culture system may well serve to model the development of human NMJ.

To our knowledge, this is the first report on simultaneous differentiation of functionally mature neurons and myotubes, and more importantly, formation of functional neuromuscular units, in cultures consisting of cells that are derived from single hPSC lines (iPSCs and ESCs). Our results are supported by a recent study showing differentiation of myotubes in parallel with neural cells from hPSCs when evaluated by using histology and gene expression analysis (Hosoyama et al., 2014). The most significant novelty of our study is the demonstration of functional NMJs derived from human iPSC and ESC lines, allowing modeling of the NMJ derived from single individuals.

Previous studies have shown quite comparable results when taking advantage of human fetal spinal cord stem cells and human muscle progenitor cells derived from satellite cells (Guo et al., 2011). In our co-culture system neural precursor cells/neurons and myogenic precursors/myotubes grow in intimate interaction with each other during the whole period of development, from the pluripotent stem cell phase until maturation of the NMJ that can be stimulated by current induction and pharmacologically. It is evident that such a long-term co-development of cells representing both presynaptic and postsynaptic cells of NMJ is needed for full maturation of NMJs. Although our co-culture model is still lacking the presence of two other components of the NMJ, Schwann cells and kranocytes (Thomson et al., 2012) the model may be useful for revealing developmental mechanisms of human NMJ as well as studying NMJ diseases.

Collectively, we demonstrate that myotubes derived from human PSC lines have excitable membrane with variety of ion conductances, mechanisms for generating muscle-cell type of calcium signals, and form functional end plates with co-differentiating neurons which are sufficient to activate myotube contraction. In a wider context, our results demonstrate for the first time that it is possible to generate two different types of cells from the same origin, which eventually form a functional connection with each other. This discovery opens up new avenues not only for cell therapy but also for tissue engineering and research of human diseases.

## Author contributions

KP, MR, and NN participated in designing the study, performed experiments, analyzed the data and wrote the manuscript; OH and PT designed experiments, analyzed data and interpreted the data and wrote the manuscript, JK planned the study, obtained funding, interpreted the data, designed a part of the study, and wrote the manuscript. All the authors worked on drafting at least parts of the work and provided critical review of the manuscript. All the authors agreed to be responsible for all aspects of the work in ensuring that questions related to the accuracy or integrity of any part of the work are appropriately investigated and resolved.

### Conflict of interest statement

The authors declare that the research was conducted in the absence of any commercial or financial relationships that could be construed as a potential conflict of interest.

## Supplementary material

Video S1 **Spontaneous myotube contractions recorded from an ESC line–derived co-culture after 6 weeks in neural differentiation medium followed by a 7-day terminal differentiation without FGF**.

Video S2 **Myoprecursors and myotubes are highly mobile, and the fusion of precursor cells into myotubes is a dynamic process revealed by a continuous live cell imaging of fixed area every 30min for 6 days at terminal differentiation phase**.
